# Accessibility and efficiency of mental health services, United Kingdom of Great Britain and Northern Ireland

**DOI:** 10.2471/BLT.20.273383

**Published:** 2021-08-17

**Authors:** Shanquan Chen, Rudolf N Cardinal

**Affiliations:** a Clifford Allbutt Building, Bay 13, Department of Psychiatry, Cambridge Biomedical Campus, University of Cambridge, Cambridge CB2 OAH, England.; b Cambridgeshire and Peterborough NHS Foundation Trust, Cambridge, England.

## Abstract

**Problem:**

Mental ill health in the United Kingdom of Great Britain and Northern Ireland has been a major driver of labour market exclusion through sickness absence, reduced productivity and job loss.

**Approach:**

A government-supported programme for improving access to psychological therapies was launched in 2008 and expanded across England in 2010. The aim was to provide evidence-based treatments for people with common mental disorders through three principal strategies: (i) routine session-by-session outcome monitoring; (ii) integration with the wider care system; and (iii) delivery of psychological therapies as part of a stepped-care approach.

**Local setting:**

Access to effective psychological therapies was previously low in the United Kingdom. In 2010, only about 35% of people with moderately severe mental disorders were in specialist or non-specialist treatment.

**Relevant changes:**

The accessibility of quality mental health services has increased, as has the efficiency of the country’s mental health system. The numbers of people entering treatment have increased steadily from 0.43 million in 2012–2013 to 1.09 million in 2018–2019. The recovery rate of patients in treatment increased from 42.8% to 52.1% during 2012–2018. The number of people moved off sick pay and benefits rose from 3683 to 18 039 over the same period.

**Lessons learnt:**

A clinical guideline on psychological therapies is a prerequisite for increasing the accessibility and efficiency of mental health services. An integrated approach allows mental health services to have better reach. Routine collection of patient-level outcome data plays an important role in the value and function of the mental health care system.

## Résumé

**Problème:**

La maladie mentale au Royaume-Uni de Grande-Bretagne et d'Irlande du Nord a été l'un des principaux facteurs d'exclusion du marché du travail à cause de l'absentéisme pour raisons de santé, de la baisse de productivité et de la perte d'emploi.

**Approche:**

Un programme d'amélioration de l'accès aux thérapies psychologiques, soutenu par le gouvernement, a été lancé en 2008 et s'est étendu à travers l'Angleterre en 2010. Le but était de proposer des traitements reposant sur des données factuelles aux personnes présentant des troubles mentaux courants, grâce à trois stratégies majeures: (i) un suivi des résultats au fil des séances; (ii) une intégration au sein du système de soins global; et, enfin, (iii) la mise en place de thérapies psychologiques dans le cadre d'une approche par paliers.

**Environnement local:**

L'accès à des thérapies psychologiques efficaces était auparavant difficile au Royaume-Uni. En 2010, seulement 35% des gens souffrant de troubles mentaux relativement graves suivaient un traitement spécialisé ou non spécialisé.

**Changements significatifs:**

L'accessibilité à des soins de santé mentale de qualité s'est améliorée, tout comme l'efficacité du système de santé mentale du pays. Le nombre d'individus ayant entamé un traitement a augmenté de façon constante, évoluant de 0,43 million en 2012–2013 à 1,09 million en 2018–2019. Le pourcentage de guérison des patients traités a grimpé de 42,8% à 52,1% entre 2012 et 2018. En outre, le nombre de personnes ayant mis fin à leurs prestations ou indemnités de maladie est passé de 3680 à 18 039 sur la même période.

**Leçons tirées:**

Une directive clinique relative aux thérapies psychologiques est l'une des conditions préalables nécessaires au développement de l'accessibilité et de l'efficacité des soins de santé mentale. Une approche intégrée permet aux services de santé mentale de toucher un plus large public. La collecte systématique des données sur les résultats observés chez les patients joue un rôle crucial dans la qualité et le fonctionnement du système de soins de santé mentale.

## Resumen

**Situación:**

En Gran Bretaña e Irlanda del Norte, las enfermedades mentales han sido uno de los principales motores de la exclusión del mercado laboral a través de las bajas por enfermedad, la reducción de la productividad y la pérdida de empleo.

**Enfoque:**

En 2008 se puso en marcha un programa apoyado por el gobierno para mejorar el acceso a las terapias psicológicas, que se amplió a toda Inglaterra en 2010. El objetivo era proporcionar tratamientos basados en la evidencia para personas con trastornos mentales comunes a través de tres estrategias principales: (i) seguimiento rutinario de los resultados sesión tras sesión; (ii) integración con el sistema de atención más amplio; y (iii) prestación de terapias psicológicas como parte de un enfoque de atención escalonada.

**Marco regional:**

Antes, el acceso a terapias psicológicas eficaces era escaso en el Reino Unido. En 2010, solo un 35% de las personas con trastornos mentales moderadamente graves recibían tratamiento especializado o no especializado.

**Cambios importantes:**

El acceso a los servicios de salud mental de calidad ha aumentado, al igual que la eficiencia del sistema de salud mental del país. Las cifras de personas que entran en tratamiento han aumentado de forma constante, pasando de 0,43 millones en 2012-2013 a 1,09 millones en 2018-2019. La tasa de recuperación de los pacientes en tratamiento aumentó del 42,8% al 52,1% durante 2012-2018. El número de personas que dejaron de recibir pagas por enfermedad y prestaciones aumentó de 3.680 a 18. 039 durante el mismo período.

**Lecciones aprendidas:**

Una guía clínica sobre terapias psicológicas es un requisito previo para aumentar la accesibilidad y la eficacia de los servicios de salud mental. Un enfoque integrado permite que los servicios de salud mental tengan un mejor alcance. La recogida rutinaria de datos de resultados a nivel de paciente desempeña un papel importante en el valor y la función del sistema de atención a la salud mental.

## ملخص

**المشكلة:**

كان اعتلال الصحة العقلية في المملكة المتحدة لبريطانيا العظمى وأيرلندا الشمالية دافعًا رئيسيًا للاستبعاد من سوق العمل نتيجة للغياب لأسباب مرضية، وانخفاض الإنتاجية، وفقدان الوظيفة.

**الأسلوب:**

تم في عام 2008 إطلاق برنامج مدعوم من جانب الحكومة لتحسين الحصول على العلاجات النفسية، وتم توسيعه عبر أنحاء إنجلترا في عام 2010. وكان الهدف منه هو توفير علاجات قائمة على الأدلة للأشخاص الذين يعانون من الاضطرابات العقلية الشائعة من خلال ثلاث استراتيجيات رئيسية: رصد النتائج بشكل روتيني جلسة بجلسة؛ و(2) التكامل مع نظام الرعاية الأكبر نطاقًا؛ و(3) طرح العلاجات النفسية جزء من أسلوب الرعاية على خطوات.

**المواقع المحلية:**

كان الحصول على العلاجات النفسية الفعالة منخفضًا في السابق في المملكة المتحدة. في عام 2010، خضع قرابة 35% فقط من الأشخاص المصابين باضطرابات عقلية متوسطة الحدة، للعلاج المتخصص أو غير المتخصص.

**التغيّرات ذات الصلة:**

زادت فرص الحصول على الخدمات الصحية العقلية ذات الجودة، وكذلك ارتفعت كفاءة نظام الصحة العقلية في الدولة. زاد عدد الأشخاص الذين يلتحقون بالعلاج بشكل ثابت من 0.43 مليون في 2012-2013 إلى 1.09 مليون في 2018-2019. كما زاد معدل شفاء المرضى نتيجة للعلاج من 42.8% إلى 52.1% خلال الفترة 2012 إلى 2018. ارتفع عدد الأشخاص الذين تركوا الرواتب والمزايا المرضية من 3680 إلى 18039 خلال نفس الفترة.

**الدروس المستفادة:**

يعد الإرشاد السريري بخصوص العلاجات النفسية متطلبًا أساسيًا لزيادة إمكانية الحصول على خدمات الصحة العقلية، وزيادة كفاءتها. يسمح الأسلوب المتكامل لخدمات الصحة العقلية بأن تنتشر بشكل أفضل. إن جمع بيانات النتائج على مستوى المريض بشكل روتيني، يلعب دورًا مهمًا في قيمة نظام رعاية الصحة العقلية، ووظيفته.

## 摘要

**问题:**

在大不列颠及北爱尔兰联合王国，心理疾病一直是导致患者遭到劳动力市场排挤的主要驱动因素，主要表现在因病缺勤、生产力下降和失业率升高。

**方法:**

在政府的支持下，2008 年推出了一项旨在普及心理治疗的计划，截至 2010 年，已将该项计划扩展至整个英格兰地区。其目的在于通过三种主要策略为存在常见心理障碍的人提供循证治疗服务：(i) 定期分工作段进行结果监测；(ii) 与更广泛的治疗系统整合；以及 (iii) 作为阶梯治疗方法的一部分，提供心理治疗。

**当地状况:**

以前，在英国获得有效心理治疗的机会很少。2010 年，仅约 35％ 的中重度心理障碍患者接受过专科或非专科治疗。

**相关变化:**

优质心理健康服务的可及性以及该国心理健康系统的效率性均得到了提高。接受治疗的人数已从 2012–2013 年的 43 万稳定增至 2018–2019 年的 109 万。在 2012–2018 年期间，接受治疗的患者的康复率已从 42.8％ 提高至 52.1％。同期，领取病假工资和津贴的人数从 3680 人增至了 18 039 人。

**经验教训:**

心理治疗相关临床指南是提高心理健康服务的可及性和效率性的先决条件。综合方法可促使心理健康服务覆盖更广的范围。例行收集患者治疗结果数据极其有利于发挥心理健康保健系统的价值和功能。

## Резюме

**Проблема:**

Состояние психического здоровья в Соединенном Королевстве Великобритании и Северной Ирландии является одним из основных факторов исключения из рынка труда из-за отсутствия по болезни, снижения производительности и потери работы.

**Подход:**

Поддерживаемая правительством программа по улучшению доступа к психологической терапии была запущена в 2008 году и распространена по всей территории Англии в 2010 году. Цель заключалась в предоставлении людям с распространенными психическими расстройствами методов лечения с научно доказанной эффективностью с помощью трех основных стратегий: (i) регулярного мониторинга результатов каждой сессии; (ii) интеграции с более широкой системой ухода; (iii) проведения психологической терапии как части поэтапного подхода к лечению.

**Местные условия:**

Ранее доступ к эффективным методам психологической терапии в Соединенном Королевстве Великобритании и Северной Ирландии был ограничен. В 2010 году только около 35% людей с умеренно тяжелыми психическими расстройствами проходили лечение как в специализированных, так и в неспециализированных медицинских учреждениях.

**Осуществленные перемены:**

Доступность качественных услуг в области психического здоровья повысилась, равно как и эффективность системы охраны психического здоровья страны. Число людей, поступающих на лечение, неуклонно росло с 0,43 миллиона в 2012–2013 годах до 1,09 миллиона в 2018–2019 годах. Показатель выздоровления пациентов, находящихся на лечении, увеличился с 42,8 до 52,1% в период с 2012 по 2018 год. За тот же период число людей, отказавшихся от выплаты пособий по болезни и пособий, выросло с 3680 до 18 039 человек.

**Выводы:**

Клиническое руководство по психологической терапии является необходимым условием для повышения доступности и эффективности услуг в области психического здоровья. Комплексный подход позволяет лучше охватить услуги в области психического здоровья. Регулярный сбор данных о результатах на уровне пациентов играет важную роль в ценности и функционировании системы охраны психического здоровья.

## Introduction

The burden of mental disorders is rising worldwide. Around one in six people suffer from mental disorders, with the highest prevalences in North America, Western Europe, North Africa, the Eastern Mediterranean and South Asia. Historically, mental health services have often been neglected and segregated from physical health; this treatment gap is bigger in low- and middle-income countries, requiring innovation and improvement.

The burden of mental ill health in the United Kingdom of Great Britain and Northern Ireland is high relative to other members of the Organisation for Economic Co-operation and Development, with mental disorders costing an estimated 70–100 billion pounds sterling (£) or 4.5% of the gross domestic product (GDP) of £1849 billion in 2010.^1^ Nevertheless, the United Kingdom has been more innovative in mental health than many other comparable countries.^1^ Here we review some lessons learnt, focusing on working-age people and illustrating improvements driven by a programme of evidence-based psychological treatment services.

## Local setting

In the United Kingdom, mental ill health has been a major factor causing labour market exclusion, through absence and reduced productivity.^1^ Almost 40% of new claims for disability benefit were made on the basis of mental ill health in 2012.^1^ However, a substantial gap existed between treatment need and treatment actually received. In 2010, only about 35% of people with moderately severe mental disorders were in treatment (specialist or non-specialist), and only about 65% of those with a severe mental disorder.^1^

The United Kingdom’s health system comprises primary care services, community-based specialist secondary care services and inpatient (secondary and tertiary) care.^2^ There are several routes to access mental health services; the most frequent is through a patient’s general practitioner in the primary care system.^3^ However, general practitioners, by definition generalists, acknowledge a lack of expertise in psychiatry.^1^ In 2005–2007, the commonest mental disorders (depression and anxiety disorders) were usually treated with antidepressants rather than psychological therapies, although the latter are cost-effective in keeping people in work or in promoting a return to work.^1^ General practitioners could refer patients to counselling services or services delivering specific psychological therapies (such as cognitive behavioural therapy), but waiting times for psychological therapies were long.^1^ A 2010 survey of 527 people found that one in five people had been waiting over a year for treatment, and one in 10 people had been waiting over two years.^1^

## Approach

The United Kingdom’s current mental health services follow the implementation of a series of programmes involving public education, workforce training and early intervention. Nevertheless, a major part of the country’s recent innovation comes from a programme called Improving Access to Psychological Therapies. In 2005, the public standard-setting body issued clinical guidelines that strongly recommended psychological therapies for the treatment of depression and anxiety disorders. Part of the subsequent economic case was that the cost of access to evidence-based psychological therapies (about £650 per patient per course) would largely pay for itself by reducing other depression- and anxiety-related public costs (such as welfare benefits and medical costs) and increasing revenues (such as taxes from a return to work).^4^ In 2005, the incoming government’s election manifesto was committed to improving mental health services, including behavioural as well as drug therapies.^4^

The new health service programme providing evidence-based psychological treatments was piloted in 2006, launched in 2008, and expanded across England in 2010 (covering 52.3 million out of the 62.5 million population in the United Kingdom).^4^ The priority was to ensure widespread access to simple, effective treatments for common mental disorders, primarily depression and anxiety disorders with mild, moderate or severe symptoms, via primary care or self-referral.^5^ The aim was therefore to address the greatest population need, rather than very severe or very complex mental disorders that are treated by secondary or tertiary mental health services. The enhanced services have three notable features.

First, routine outcome monitoring is conducted on a session-by-session basis by the therapy service,^5^ with 98% of patients having self-reported symptom scores recorded at the beginning and end of treatment.^6^ Data are also collected on the person’s disability (how their mental health problem interferes with their functioning in work and normal life); their employment; and their experience of the quality of care received. Summary data are made publicly available. Patients can see what their local mental health therapy service oﬀers and the outcomes it achieves; service commissioners and clinicians can benchmark their service against others and develop collaborative networks for services to learn from each other.^6^ Meanwhile, session-by-session data enable clinical monitoring and supervision, using objective measures of effectiveness.^5^ Local, regional and national leaders use these data for policy-making.^6^

Second, the programme provides evidence-based psychological therapies via a stepped-care approach (Box 1).^5^ Therapy can be delivered by a single clinician, with or without concurrent medication management (usually by a general practitioner).

Box 1 **Stepped-care services in the Improving Access to Psychological Therapies programme, United Kingdom of Great Britain and Northern Ireland**

Step 1. Primary care, usually provided by a general practitioner: includes identification and assessment of problems (including risk assessment); psychoeducation; active monitoring; and referral for further assessment and interventions.

Step 2. Low-intensity service, often provided by a psychological well-being practitioner: mainly offers low-intensity interventions, including guided self-help; computerized cognitive behavioural therapy; and group-based physical activity programmes. Targeted at patients with sub-threshold depressive symptoms and mild-to-moderate depression, as well as some people with anxiety disorders.

Step 3. High-intensity service: usually involves weekly face-to-face, one-to-one sessions with a trained therapist. Also includes cognitive behavioural therapy group work or couple therapy for depression.

Sources: National Collaborating Centre for Mental Health, 2018;^5^ National Institute for Health and Care Excellence, 2011.^7^

**Fig. 1:**
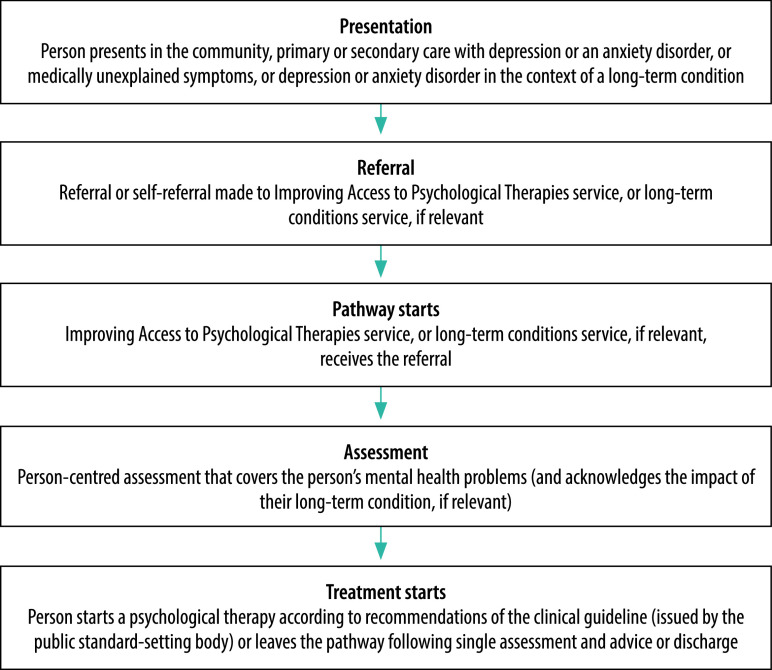
Pathway for Improving Access to Psychological Therapies services, United Kingdom of Great Britain and Northern Ireland

Source: We created the figure using information from a service manual and clinical pathway description from the National Collaborating Centre for Mental Health.^5^^,^^9^

Third, services are integrated with the wider care system beyond mental health, such as social and physical health care, and across the lifespan (education and employment).^5^ For instance, social prescribing is seen as supporting delivery of the enhanced services.^5^ Social prescribing includes facilitated self-help, personal skills development and book therapy, with some components available online. These services may increase support for patients, particularly at an early stage of illness, although the evidence base remains weak.^8^ Patients with comorbid long-term physical health conditions or medically unexplained symptoms may be referred into a focused pathway (Fig. 1).^9^ The programme provides specific assessment and treatment protocols for students.^5^ Employment advice has been integrated into the services and delivers a personalized service to patients based on their individual needs.^1^^,^^5^

## Relevant changes

Patients treated and measures of the programme’s clinical success have increased steadily in England over the period 2012–2013 to 2018–2019 (Table 1). The numbers of patients referred to the programme’s services almost doubled from 0.88 million to 1.60 million. The numbers of patients entering treatment rose from 0.43 million to 1.09 million and the numbers finishing treatment rose from 0.14 million to 0.58 million. The proportion of patients referred who started treatment within 4 weeks increased from 63.3% (274 975/434 274) to 78.2% (853 880/1 090 296). Outcomes of treatment also improved over the study period. The percentage of patients whose condition improved rose from 57.5% (82 910/144 210) to 67.4% (392 662/582 556) and the proportion recovering rose from 42.8% (54 430/127 060) to 52.1% (284 810/546 660). Overall employment metrics have improved, possibly related to programme expansion or starting therapy earlier when people still have a job (Table 1). Over time, more employees accessed the programme, as did the number who moved off sick pay or benefits (rising almost fivefold, from 3683 people in 2012–2013 to 18 039 people in 2018–2019). We cannot assume causality, however, as other workplace factors may also have played a role.

**Table 1:** Activity, waiting times and outcomes in the Improving Access to Psychological Therapies programme, United Kingdom of Great Britain and Northern Ireland, 2012–2018

Variable	Year						
	2012–2013	2013–2014	2014–2015	2015–2016	2016–2017	2017–2018	2018–2019
**Activity^a^**							
No. of patient referrals received, millions	0.88	1.12	1.27	1.40	1.39	1.44	1.60
No. of referrals where patient entered treatment, millions	0.43	0.71	0.82	0.95	0.97	1.01	1.09
No. of referrals where patient finished treatment, millions	0.14	0.36	0.47	0.54	0.57	0.55	0.58
Mean no. of treatment appointments per finished course of treatment^b^	NR	NR	6.30	6.40	6.60	6.80	6.90
**Waiting times^a^**							
No. (%) of referrals where patient started treatment within 4 weeks, millions^c^	0.27 (63.3)	0.44 (61.4)	0.55 (66.9)	0.71 (74.3)	0.76 (78.8)	0.80 (79.6)	0.85 (78.2)
**Outcome^a^**							
No. (%) of referrals with reliable improvement of patient, millions^d^	0.08 (57.5)	0.22 (59.7)	0.29 (60.8)	0.33 (62.2)	0.37 (65.1)	0.37 (66.4)	0.39 (67.4)
No. (%) of referrals with recovery of patient, millions^e^	0.05 (42.8)	0.14 (45.0)	0.19 (44.9)	0.23 (46.3)	0.26 (49.3)	0.26 (50.8)	0.28 (52.1)
No. (%) of referrals with reliable recovery of patient, millions^e^	0.05 (40.9)	0.14 (42.8)	0.18 (42.8)	0.22 (44.0)	0.25 (47.0)	0.25 (48.3)	0.27 (49.5)
**Employment support**							
No. of referrals where patient finished a course of treatment and was in employment at the start, millions	0.07	0.18	0.24	0.28	0.31	0.32	0.34
No. of referrals where patient finished a course of treatment and was in employment at the end, millions	0.07	0.17	0.23	0.27	0.29	0.30	0.33
No. (%) of referrals where patient finished a course of treatment and was employed at the start and end, millions^f^	0.06 (43.9)	0.15 (42.0)	0.21 (43.9)	0.24 (45.3)	0.27 (47.5)	0.28 (49.8)	0.30 (51.1)
No. (%) of referrals where patient moved off sick pay and benefits^f^	3683 (2.6)	10 982 (3.0)	15 312 (3.3)	17 925 (3.3)	18 628 (3.3)	17 779 (3.2)	18 039 (3.1)

^a^ Indicators used in the United Kingdom’s National Health Service’s annual report. Referrals are the primary entity being counted. A referral constitutes an episode of care for a patient (it is possible for a single patient to be referred more than once).

^b^ Appointments are the way in which patients’ contact with services are recorded.

^c^ The denominator is the number of referrals where the patient entered treatment.

^d^ A patient has shown reliable improvement if they have a significant improvement in their condition following a course of treatment. Improvement is measured by the difference between their first and last scores on questionnaires tailored to their specific condition. The denominator is the number of referrals where the patient completed treatment.

^e^ Recovery in the programme is measured in terms of caseness, a term which means a referred patient has severe enough symptoms of anxiety or depression to be regarded as a clinical case. A referred patient has moved to recovery if they were a case at the start of their treatment, and not by the end of their treatment, as measured by scores from questionnaires tailored to their specific condition. A referred patient has reliably recovered if they meet the criteria for both the recovery and reliable improvement measures. The denominator is the number of referrals for patients who completed treatment minus those not at caseness.

^f^ The denominator is the number of referrals where the patient finished a course of treatment.

NR: not recorded.

Notes: We extracted data from the annual report on the use of Improving Access to Psychological Therapies services for England.^10^ State-provided health care is run separately in the four constituent countries of the United Kingdom, of which England is one. Taking 2018–2019 as an example, the data were collected from 1 April 2018 to 31 March 2019.

An economic evaluation of the associated employment support services in 2011 estimated that every £1 spent generated £2–3 in GDP, of which 30% benefited the individual and 70% the state.^11^ For those patients with long-term physical conditions, early treatment was associated with substantial savings in hospital costs during the subsequent year, and these savings broadly covered the cost of the mental health treatment, ranging from over £400 saved for patients with comorbid diabetes to over £800 saved for patients with comorbid chronic lung and cardiovascular diseases.^12^

## Lessons learnt

In the United Kingdom, progress has been made towards the World Health Organization (WHO) 2013–2020 mental health goals, which include providing good physical and mental health care for all, mental health systems working with other sectors, and mental health governance and delivery via good information. The United Kingdom experience resonates with the WHO–Gulbenkian Global Mental Health Platform’s strategies to improve mental health systems by adopting evidence-based practice, considering the whole life course, and enhancing inter-sector cooperation. The Improving Access to Psychological Therapies programme has been criticized for using predominantly self-report measures and for inconsistent provision of relapse prevention support,^13^^,^^14^ while rates of improvement are not equal for all patient groups.^14^ Nevertheless, the programme’s experience may prove useful for other nations, even in resource-poor settings (Box 2).

Box 2 **Summary of main lessons learnt**

- A clinical guideline on psychological therapies is a prerequisite for increasing the accessibility and efficiency of mental health services.
- An integrated approach can increase the reach of mental health services.
- Patient-level routine outcome data play an important role in individual care and the performance of the mental health care system.

First, clinical guidelines are a prerequisite for increasing the accessibility and efficiency of mental health services. National guidelines enabled large-scale training in evidence-based therapies, mitigating early concerns about staffing requirements. The programme recommends systematic screening for every condition that it treats.^5^ This process is applicable to resource-poor settings without electronic information systems, using simple paper-based tools. While access to psychotropic medicines is important, the widespread provision of psychological therapies provides an additional treatment approach in settings with limited access to psychotropics.

Second, an integrated approach allows mental health services to have better reach. The vertical integration approach corresponds to the standard-setting body’s stepped-care model, with broad access to treatments for common mental disorders in primary care, and patients being stepped up, including to secondary care if required, in complex or high-risk situations or following treatment failure. Horizontal integration of mental health services within a wider care system – including social care, physical health care and education and employment – increases the possibility to intervene early, especially for some at-risk groups. Although integration of services depends on the United Kingdom’s well-developed information system, the idea or mechanism behind it could also be applied in resource-poor settings. Both vertical and horizontal integration depend on the foundations of trained personnel and a standardized diagnostic process, as well as the initial detection and referral of patients from primary care or opportunities for self-referral.

Third, quantitative outcome measures support high-quality mental health care. While changes in self-report symptom measures do not preclude bias, treatments in the Improving Access to Psychological Therapies programme are based on randomized controlled trial evidence^15^ and strong evidence for efficacy from a meta-analysis.^13^ Collecting patient-level outcomes routinely promotes individual clinical care (“is my patient getting better?”), clinical supervision, and service quality improvement (“how is my local service performing compared with others?”).^6^ These methods are most powerful in the context of electronic information systems, but remain applicable in resource-poor settings.
